# Using Semantic Web Technologies to Annotate and Align Microarray Designs

**DOI:** 10.4137/cin.s2335

**Published:** 2009-05-13

**Authors:** Sebastian Szpakowski, James McCusker, Michael Krauthammer

**Affiliations:** 1 Program for Computational Biology and Bioinformatics (CBB), Yale University School of Medicine, New Haven, CT.; 2 Department of Pathology, Yale University School of Medicine, New Haven, CT.

**Keywords:** semantic web, ontology, genomics, data integration, annotation

## Abstract

In this paper, we annotate and align two different gene expression microarray designs using the Genomic ELement Ontology (GELO). GELO is a new ontology that leverages an existing community resource, Sequence Ontology (SO), to create views of genomically-aligned data in a semantic web environment. We start the process by mapping array probes to genomic coordinates. The coordinates represent an implicit link between the probes and multiple genomic elements, such as genes, transcripts, miRNA, and repetitive elements, which are represented using concepts in SO. We then use the RDF Query Language (SPARQL) to create explicit links between the probes and the elements. We show how the approach allows us to easily determine the element coverage and genomic overlap of the two array designs. We believe that the method will ultimately be useful for integration of cancer data across multiple *omic* studies. The ontology and other materials described in this paper are available at http://krauthammerlab.med.yale.edu/wiki/Gelo.

## Introduction and Background

The sequencing of the human genome^1^,^2^ and subsequent annotation initiatives^3^,^4^ are creating a large body of information on genome accessibility (methylation and histone modifications), transcription (mRNA, ncRNA expression), and structural variations (such as inversion, duplication and translocation).^5^–^7^ The task of organizing such large volumes of data becomes increasingly complex, as do the subsequent analyses of the information.^8^–^11^ In an effort to catalog this and similar information, well over 1,000 different databases are currently actively maintained in the realm of molecular biology.^12^ The problem is that many of them are neither connected nor integrated.^8^–^10^,^13^

The area of data integration using semantic web technologies remains under active development.^13^–^17^ Compared to more traditional relational database systems, the use of semantic web technologies simplifies data integration through W3C-supported knowledge representation standards such as Resource Description Framework Schema,^18^ and Web Ontology Language.^19^ A growing list of standardized vocabularies and data sources in RDFS and OWL, such as Gene Ontology (GO),^20^ Sequence Ontology (SO),^21^ and other projects within the realm of Open Biological Ontologies (OBO),^22^ allow the scientific community to move away from a plethora of home-built data models towards a situation where numerous data and knowledge bases share the same or related upper level schemas. This standardization of data models is desirable to facilitate the sharing of cancer data across multiple genomic data stores. Also, in the area of human genomics, where new facts and types of facts are discovered on a regular basis, a traditional relational model of storing data becomes less than optimal. For example, to include a new type of fact, a rigidly defined relational-database table would need to be updated with additional columns to accommodate the new information. In contrast, triple stores can easily add new properties to existing information by means of subject-predicate-object triples. Finally, an additional benefit of using semantic web technologies, albeit currently underutilized, involves the possibility of implementing reasoners that can logically infer relationships among the entities in the store. Triple stores allow for queries that are not easily performed in traditional databases, such as queries across hierarchies, as in ontologies. Reasoner software can also help in performing consistency checks over complex knowledge bases using logical rules.^8^

In this study we discuss the use of semantic web technology for array annotation and alignment. Most of our data is derived from cancer microarray experiments. A critical step in the microarray data integration process is the alignment of the different microarray designs to perform integrated analyses. Mapping of array probes to genomic coordinates is essential for this task. The coordinates represent an implicit link between the probes and multiple genomic elements such as genes, transcripts, miRNA, and repetitive elements which are annotated using concepts from Sequence Ontology (SO).^21^ By creating explicit links between the loci of genomic elements, we are able to derive which probes and elements align. The mapping of probes to elements achieves two goals: first, it links probes to gene transcripts (elements in SO), allowing for the re-annotation of the array design with the transcripts covered. Second, we can establish the overlap between the probes of two different array designs, establishing the degree of alignment.

## Materials and Methods

### GELO

Our project provides a unique approach to linking various data in the area of molecular biology using semantic web technologies. Unlike other approaches, such as Bio2RDF^14^ that rely on database identifiers, names, and synonyms to link information, we use the genomic coordinates as a biologically-meaningful scaffold to attach and align information. Creating synonyms to link disparate sources of data is a useful approach but it requires time-consuming manual curation. Our approach allows the system to automatically infer that any two elements are the same if they map to the same coordinates in a particular genome build. Consequently, any annotation pertinent to one element can be applied to the other as well. Additionally, each genomic element can be automatically represented in the context of other elements by means of relationships such as “***a*** *upstream_of* ***b***”, “***c*** *on_the_same_strand_as* ***d*** ”, or “***e*** *contains* ***f*** ”. This allows for complex queries such as for exons that are contained in a particular transcript.

**Figure 1:**
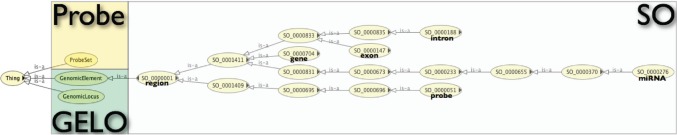
A graph representing the classes of Sequence Ontology (SO), GELO and PROBE. Only a subset of SO classes are shown. Labels correspond to SO’s label property for nodes.

Our Genomic ELement Ontology (GELO) (Table 1 and Fig. 1) is loosely based on an Open Biological Ontology^22^ project called Sequence Ontology (SO),^21^ a standard for annotating regions of the human genome. ***“Region”***, an (incomplete) sub-branch of SO, is used as the basis of the GELO ontology. Our **“*GenomicElement*”**, a superclass of SO’s **“*region*”**, subsumes all terms predefined in SO (*e.g.* **“*repetitive element*”**, **“*ncRNA*”**, etc.). The class **“*GenomicElement*”** is flexible enough, however, so that it could be used to conceptually represent any of the following: **“*the entire genome*”**, **“*a single chromosome*”**, **“*a band on a chromosome*”**, **“*an n-megabase-long region*”**, **“*a specific gene*”**, **“*an exon*”**, *or* **“*a unique 50-mer within the exon*”**. A novel class **“*GenomicLocus*”** was created to provide a facility to link any **“*GenomicElement*”** to its sequence in a particular assembly of the human genome (Table 1). The relationships “*locus_of*” and “*has_locus*” were defined to link **“*GenomicElement*”** with its biological coordinates stored in a **“*GenomicLocus*”**. To describe the relative position of two instances of **“*GenomicLocus*”** in the genome, “*contains*” and “*contained_by*” (subclasses of “*has_part*” and “*part_of*”, already defined in SO) were defined in GELO. Several other properties will be defined to facilitate the relative positioning of the regions: transitive “*upstream_of*” and its inverse: “*downstream_of*”, symmetric “*on_the_same_strand_as*” with an analogous “*on_the_opposite_strand_from*”, symmetric “*overlaps_with*”, and so on. The proof of concept described in this manuscript relies only on the relationships “*contains*” and “*contained_by*”. As our repository grows, other relationships not discussed in this manuscript will be added as well.

### Knowledge base

We describe the process used to construct our knowledge base using GELO and a set of genomic sequences. Two sets of sequences were used, both being lists of probes from commercial Nimblegen microarrays. The two sets indicate an evolution of the microarray design as the first one was generated in 2005 (2005-04-20_Human_60mer_1in2 array) and the second in 2006 (2006-08-03_HG18_60mer_expr). Both sets of probes are available within Nimblegen design files. The first step was to take the sequences of the probes and strip them of any existing annotation. The next step involved mapping the sequences to the most current build of the human genome (hg18,)^23^ using the BLAST-Like Alignment Tool (BLAT).^24^ We set BLAT to find probe alignments with 50% and better similarity scores. A resulting PSL file was then parsed using a custom python script, which converted the tabular format into Subject-Predicate-Object triples, stored in the N-Triples format.^25^ The N-Triples format was chosen because of its simplicity in comparison to other formats such as RDF/XML,^26^ Turtle^27^ or N3.^28^ Within the N-Triples representation, all sequences have been represented as individuals of *RDF: type* “***probe***” (defined in SO as “***SO_0000051***”) and all BLAT matches were represented as of *RDF: type* “***GenomicLocus***” annotated with genomic coordinates found by BLAT. All “***GenomicLocus***” individuals were linked to their appropriate ***“probe”*** individuals using “*locus_of*” relationship from GELO. To ease the retrieval of probe locations from one design file over others, the concept “***ProbeSet***” was defined in a separate (helper) ontology called Probe (Table 1). Two instances of “***ProbeSet***” were created, one aggregating probe sequences from the 2005 design file, the other aggregating probe sequences from the 2006 design file. The “*part_of*” relationship was used to link particular probes with their corresponding probe design.

At the time of publication our knowledge base contained information about genes, their transcripts, and the locations of introns and exons. The import of gene information was performed as follows. A list of FASTA files containing the sequences of all human transcripts was acquired from the Refseq database at NCBI (RefSeq release 33).^29^ The information linking the genes with gene names, gene symbols, synonyms, *etc*, was acquired from two files: homo_sapiens.gene_info and gene2refseq (both available via FTP from NCBI’s gene database).^30^ The sequences were aligned with the latest build of the human genome (hg18)^23^ using BLAT.^24^ Subsequently the tabular output files of BLAT together with transcript and gene annotation from NCBI were converted to N-Triples representation using a custom python script.

Within the N-Triples representation all known genes were defined as *rdf: type* “***SO_0000704***” and all known transcripts using *rdf: type* “***SO_0000673***” (both defined in SO). All BLAT matches were represented using *rdf: type* “***GenomicLocus***” defined in GELO. Finally all gene individuals were linked to their respective transcripts using “*has_transcript*” relationship (using an ENTREZ helper ontology created by our group to augment GELO with gene-specific relationships, see Table 1), and all transcripts were linked to their appropriate “***GenomicLocus***” (i.e. BLAT mappings) using the “*has_locus*” relationship defined in GELO. Thus, an indirect link from genes to their respective locations was achieved. As every line of the psl file contains information about “block start” and “block end,” indicative of the intron-exon structure of the transcript, this information was also included in the N-Triples file, whereas the introns were created as SO-defined “***SO_0000188***” instances and exons as instances of “***SO_0000147***”. Each intron and exon were linked to their respective BLAT-determined “***GenomicLocus***” instances via the “*has_locus*” relationship.

We decided to use BigOWLIM as our storage system based on published and unpublished LUBM Benchmarks.^31^ Additionally, BigOWLIM uses the Sesame API which was successfully used in other semantic web projects.^32^–^34^

After loading all elements (probes, genes, introns, exons) and their respective loci on the chromosomes, we needed to determine which elements’ loci overlap along the chromosome. We defined an “***a*** *contains* ***b***” semantic relationship as a relationship between any two individuals of class “***GenomicLocus***” such that an entire genomic sequence of an individual ***b*** can be found within the sequence of an individual ***a***. As we were mainly interested in exploring the short 60-mer probe sequences in the context of their belonging to relatively long transcript sequences, we focused on the “*contains*” relationship only. Other relationships, such as “*overlaps*”, although equally important, were assigned a lower priority and will be added to the repository in the future.

**Figure 2:**
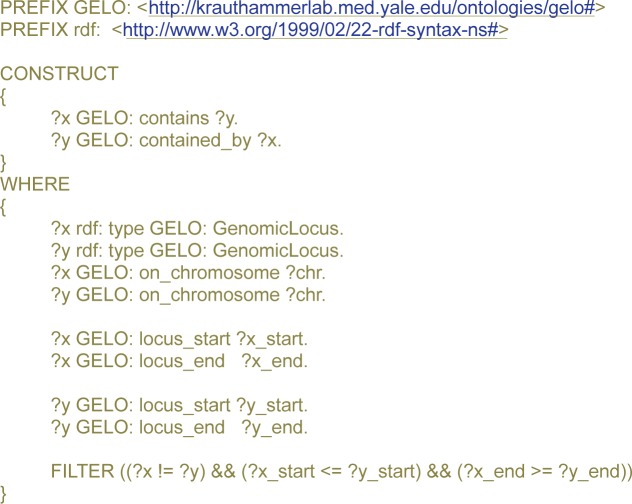
SPARQL query for finding ***GenomicLocus*** instances x and y, such that x contains ***GenomicLocus*** y.

To potentially link two loci using the “*contains*” relationship, the knowledge base was queried using a SPARQL^35^ expression (Fig. 2) to construct a new graph linking pairs of loci. The rule engine of OWLIM allows to create logic rules equivalent to the SPARQL expression listed in Figure 2. Currently, OWLIM rules do not support “bigger than” or “smaller than” constraints, but future versions will do so (Personal Communication). The idea is that the rule engine will infer the “*contains*” and other relationships of GELO automatically upon insertion of new “***GenomicLocus***” data into the knowledge base. At this time, we resorted to constructing sub-graphs using SPARQL queries.

**Figure 3:**
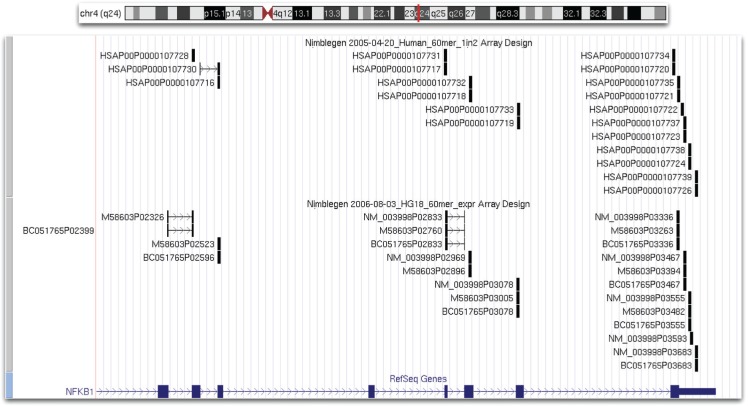
Probes from Nimblegen 2005-04-20_Human_60mer_1in2 and 2006-08-03_HG18_60mer_expr array designs that map to the NFκB1 locus. Duplicate probes with the same position share the same sequence, but were assigned to separate nucleotide sequence accessions in the original design files.

A simple validation of the repository was done by probing the genomic vicinity of the NFκB1 gene. A query was issued to retrieve all probes and their locations for all known transcripts of the NFκB1 gene. Figure 3 shows the region with all probes retrieved plotted using the UCSC genome browser.^24^ A comparison between 1) a list of probes that was originally associated with the gene during the array design process, and 2) the list of probes retrieved from our repository showed the following: first, our repository correctly mapped all NFκB1 probes from the 2005 and 2006 designs to the NFκB1 locus. Second, our list contained two extra probes over the original design. Further examination of the extra probes revealed them to be probes designed for other genes that imperfectly matched the NFκB1 locus. Adjusting the threshold of “***blat_match_score***” removed the imperfect BLAT matches.

The ontologies described in this paper can be accessed at http://krauthammerlab.med.yale.edu/wiki/Gelo.

## Results and Discussion

The goal of our knowledge base is the alignment of genomic data from cancer high-throughput experiments. We currently work with melanoma gene expression data from two different array designs, and we are interested in aligning the results of both designs. Having constructed our knowledge base of genes and their genomic locations, we attempted to re-annotate the sequences of the two microarray designs, the 2005 design (2005-04-20_Human_60mer_1in2) with 383,468 probe sequences, and the 2006 design (2006-08-03_HG18_60mer_expr) with 381,002 probe sequences, and to determine the genomic overlap.

**Figure 4:**
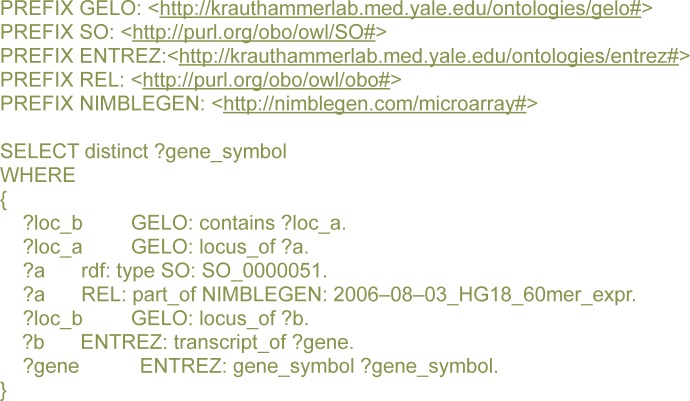
A sample SPARQL query displaying a list of genes with transcripts containing at least one probe.

Figure 4 shows the SPARQL query used to align the two design files and determine how many individual transcripts and genes are probed in each of the designs.

**Figure 5:**
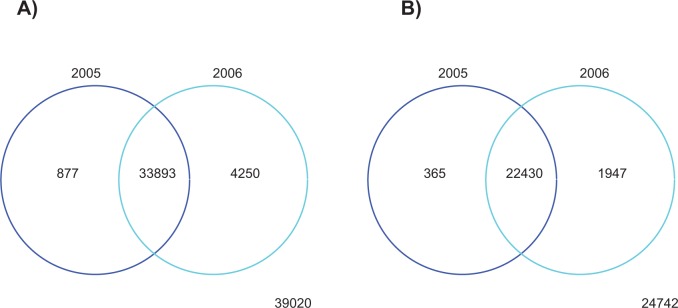
Differences among the two microarray design files. **A**) Number of transcripts that contain at least one probe. **B**) Number of genes with a transcript that contains at least one probe.

The Venn diagram in Figure 5 illustrates the query result. Not surprisingly, the 2006 design features 1947 new genes that were not included in the previous year’s design (to produce the graph from the results of the SPARQL query, we used Python and R). A further examination of the genomic overlap among the two design files revealed 365 genes that were not included in the newer design. The differences among the design files could reflect the changes in the assembly of the human genome sequence as well as changes in the annotation of the sequences provided by Refseq.^29^ The alignment of the two design files will enable user to determine which of the gene-specific probe sets can be compared between the two different designs.

**Figure 6:**
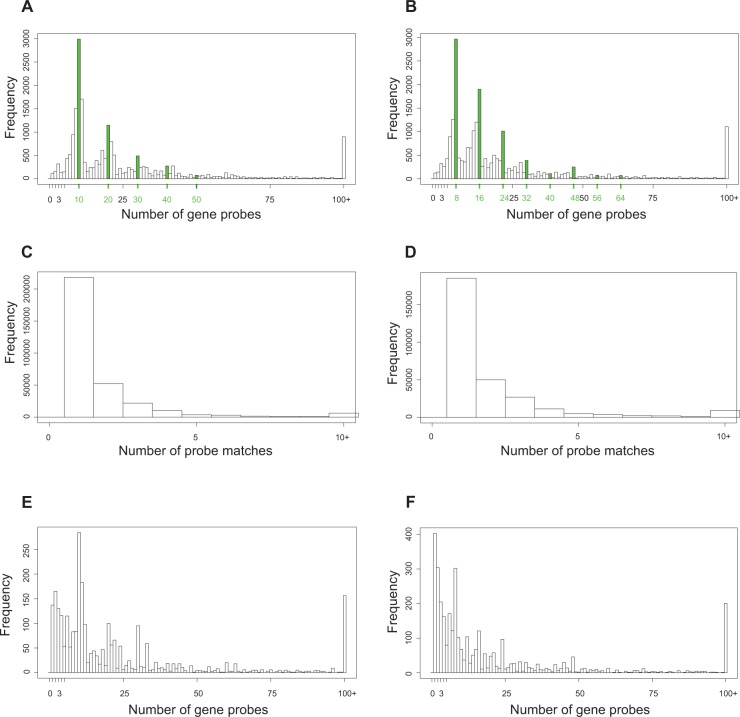
Relationship between genes and probes. **A**) 2005 design, number of probes *contained*_*by* each gene where both gene and probe are located on the same strand of DNA. The highlight in green indicates a strategy to design 10–11 probes per gene. **B**) 2006 design, number of probes *contained_by* each gene where both gene and probe are located on the same strand of DNA. The highlight in green indicates a strategy to design 8 probes per gene. **C**) 2005 design, number of genes probed by each probe, both gene and probe are located on the same strand of DNA. **D**) 2006 design, number of genes probed by each probe, both gene and probe are located on the same strand of DNA. **E**) analogous to A, the probe and the gene are located on the opposite strands of DNA, **F**) analogous to B, the probe and the gene are located on the opposite strand of DNA.

Next, we investigated the number of probes that are contained within each of the genes. An overwhelming majority of the genes had 10 probe sequences assigned to them in the 2005 design and 8 for the 2006 design. This agreed with the prior knowledge about these microarray designs. The histograms in Figures 6A and 6B revealed a periodicity in probe count distribution. For example, there are several genes for which 20, 30, etc., probes were selected in the 2006 design. This is a reflection of the number of transcripts covered per gene. The question is whether some of these probes are duplicates. To address this question, we investigated one of the genes, NFκB1, which had a probe count corresponding to two transcripts. The illustration in Figure 3 shows the NFκB1 locus and the different array probes. It is evident that quite a few of the probes overlap: ~7734 and ~7720, ~7735 and ~7721, and so on. A further examination of the repository revealed that NFκB1 is linked to two (2005 design) and three (2006 design) uniquely identified, although completely identical, transcripts. The probe sequences were selected for each of the transcripts independently, possibly without acknowledging that they were, in fact, the same. As a result, in the 2005 design, we observe duplicates of certain probes, and in the 2006 design, triplicates of probes. Querying our knowledge base revealed that several different locations on the microarray surface store the same probe sequence. A researcher can use the information provided by the knowledge base and compare the microarray surface locations storing the same probe sequence to detect variability in the microarray data.

The alignment of the two design files can now be used to revise and supplement the incomplete annotation of the original design files. Specifically, a closer look at the original annotation included in the 2006 design revealed that probes were designed for 41,621 unique transcripts identified by either Refseq ids (20,590 transcript) or GenBank accession ids (21,031 transcripts). Unlike the GenBank accession ids, the Refseq ids correspond to well-curated consensus messenger RNA sequences. A query to our knowledge base showed that probes which were originally mapped to the 20,590 Refseq sequences are re-mapped to 24,079 Refseq ids. The 21,031 transcripts with GenBank accessions ids are re-mapped to 17,332 refseq transcripts. Overall, the 41,621 transcript ids were re-mapped to 24,644 unique Refseq ids (versus 20,590 in the original design) based on probe sequence alignment to the human genome.

Figures 6C and 6D show two histograms depicting the uniqueness of probes with respect to genes. As expected, the majority of the probes in both 2005 and 2006 designs are unique, i.e. they report on expression level of just one sequence. However, the skew of the distributions suggests the presence of many “noisy” probes whose sequences match more than one, and sometimes even more than two or three genes. The re-annotation of the 2005 and 2006 design files can report on how “promiscuous” any given probe is, which is useful for signal normalization and de-noising of the microarray data.

Another aspect of our knowledge base is the inclusion of information describing the polarity of probe and gene sequences. Figures 6E and 6F show the presence of probes in gene regions where the probes are on the opposite strand from the gene. The polarity of probe sequences with respect to gene sequences may be relevant for some experimental designs. Alternatively, in an experimental design where the relative position of the probe should not matter, the repository can be queried to find additional probes that, although anti-sense with respect to the gene, can be examined to further strengthen the evidence coming from the other, correct-sense probes.

We would also like to discuss the performance of the knowledge base, which currently stores over 39,000,000 explicit statements (triples). The query in Figure 4 took 82,863 ms (1.45 min) to complete and returned 1,253,878 statements mapping probes and transcripts for the 2005 design. The same query for the 2006 design completed within 102,000 ms (1.7 min) with 1,714,030 statements returned. A more complex query to return all probes mapped to the NFκB1 gene (Fig. 3) took 411,692 ms (6.86 min) to return 41 statements. Semantic web technologies are evolving, and the time it takes to complete the queries will surely decrease in the future. For the purpose of our research, however, the response time was satisfactory.

Overall, our knowledge base provides a biologically meaningful framework for the examination of genomic data. The potential of the semantic web to link virtually any piece of information in the context of its genomic location provides an attractive strategy for data integration and analysis in the 21st century.

## Figures and Tables

**Table 1:** GELO, ENTREZ and PROBE helper ontologies. The three ontologies link to classes in sequence Ontology. The helper ontologies define 3 new classes, highlighted in bold.

Ontology	Domain	Relationship	Range/Type
GELO	**GELO:GenomicElement**	rdf:type	owl:class
	**GELO:GenomicElement**	GELO:has_locus	**GELO:GenomicLocus**
	**GELO:GenomicElement**	SO:has_part	**GELO:GenomicElement**
	**GELO:GenomicLocus**	rdf:type	owl:class
	**GELO:GenomicLocus**	GELO:start	Integer
	**GELO:GenomicLocus**	GELO:end	Integer
	**GELO:GenomicLocus**	GELO:strand	+/−/*
	**GELO:GenomicLocus**	GELO:on_chromosome	SO:Chromosome
	**GELO:GenomicLocus**	GELO:in_assembly	SO:Assembly
	**GELO:GenomicLocus**	GELO:locus_of	**GELO:GenomicElement**
ENTREZ	SO:Gene	ENTREZ:has_transcript	SO:Transcript
	SO:Gene	ENTREZ:gene_symbol	string
	SO:Gene	ENTREZ:synonym	string
	SO:Gene	ENTREZ:entrez_gene_id	string
	SO:Gene	ENTREZ:description	string
	SO:Gene	ENTREZ:taxonomy_id	string
	SO:transcript	ENTREZ:transcript_of	SO:Gene
	SO:transcript	ENTREZ:RNA_nucleotide_accession	string
PROBE	**PROBE:ProbeSet**	rdf:type	owl:class
	**PROBE:ProbeSet**	SO:has_part	SO:Probe
SO	SO:Region	rdf:subclass_of	**GELO:GenomicElement**
	SO:Gene	rdf:subclass_of	SO:Region
	SO:Transcript	rdf:subclass_of	SO:Region
	SO:Intron	rdf:subclass_of	SO:Region
	SO:Exon	rdf:subclass_of	SO:Region
	SO:miRNA	rdf:subclass_of	SO:Region
	SO:Probe	rdf:subclass_of	SO:Region
